# Health promotion perspectives on the COVID-19 pandemic: The importance of religion

**DOI:** 10.1177/1757975920972992

**Published:** 2020-11-23

**Authors:** Sima Barmania, Michael J. Reiss

**Affiliations:** UCL Institute of Education, London, United Kingdom

**Keywords:** religion, COVID-19, public health, health promotion, complexity theory, sensitivity

## Abstract

In this article we examine the importance of religion for COVID-19 health promotion. We advance three main arguments. First, religion plays an important role in affecting how likely it is that people will become infected with COVID-19. Second, religion should not be seen as a ‘problem’ with regards to COVID-19 but as an important part of the worldview and lifestyle of many people. Third, there are valuable health promotion lessons we can learn not only from the intersection of religion and other infectious diseases, but also from approaches taken within science education. Contentious science topics such as evolution and vaccine hesitancy have been effectively communicated to those with a religious faith who are disposed to reject them by reframing and considering religion as a worldview and treating those who do not accept standard scientific theories sensitively. Religion has much to contribute to health promotion, including introducing perspectives on life’s meaning and on death that can differ from those held by many without religious faith. Furthermore, religious leaders are important gatekeepers to their communities and can therefore play a vital role in policy implementation, even when that policy makes no overt reference to religion. Our contention is that by working with those of faith in the context of COVID-19, health promotion can be enhanced.

## Introduction

Since the first identified case of COVID-19 was discovered in Wuhan, China at the end of 2019 (1) there has been an unprecedented interest in public health across all sectors. In an extraordinarily short timeframe, there has been an outpouring of scientific research on the disease’s origins, zoonotics, pathophysiology and clinical outcomes (2). Epidemiological research and modelling have revealed important findings, such as incubation period and infectivity patterns, which have become cornerstones in public health policies to mitigate the spread of COVID-19. Physical distancing, face masks and regular handwashing are practices that have all been employed to decrease the risk. Within individual nations there have been studies to identify which specific groups are disproportionately affected (3).

However, such understandings alone are not sufficient to respond to the epidemic in ways that are both effective and efficient. There are benefits to a more holistic approach to understanding health behaviours and the social and cultural settings which mitigate or exacerbate health interventions and the multifaceted nature in which such interventions take place (4). There would also be benefits from those working in public health rooting their work within a framework that recognises the importance and context of social settings. Given the utility of a more holistic approach that acknowledges the significance of context, a principal argument of this article is that the importance of religion for COVID-19 is currently underappreciated. Although Dahlgren and Whitehead’s (5) model on the social determinants of health does include economic factors, ethnicity and culture, it makes no reference to religion. This is despite the fact that, to a greater or lesser degree, religion around the globe affects the large majority of people’s daily lives and social activities – often influencing their health, either directly or indirectly (6). Likewise, other health models often do not mention religion or traditional beliefs. Cohn and colleagues’ (7) model, with its description of complexity as ‘a dynamic and constantly emerging set of processes and objects that not only interact with each other, but come to be defined by those interactions’ (p.42), is particularly applicable to the current COVID-19 situation; religion certainly contributes to this complexity.

In this article we therefore examine how religion is important for COVID-19 health promotion by first looking at how religion plays an important role in determining how likely it is that someone will get infected with COVID-19, and then consider how religion is often depicted as a problem for health promotion. We then draw on both complexity theory and approaches to dealing with religion in science education to suggest how religious leaders can help in the ‘fight’ against COVID-19, and conclude with a wider discussion around religion and health promotion within the context of COVID-19.

## Religion and COVID-19 infection

Religion was first brought to public notice in relation to COVID-19 at the start of 2020 when a number of cases erupted amongst those who visited Iran’s Shia Muslim holy sites of Qom and Mashhad in February, and who subsequently travelled within the Middle East (8). In South Korea, the origin of thousands of cases of COVID-19 is thought to have been the Shincheonji Church of Jesus (9). In Southeast Asia, a 14,000 strong delegation of Islamic Tablighi-Jamaat in Kuala Lumpur was widely considered to be the cause of the second wave of the pandemic in Malaysia, with attendees from the event travelling to Brunei, Cambodia and Indonesia and later testing positive (10).

The significance of religious congregation is advocated within both Abrahamic and non-Abrahamic religions (11). Such congregation may entail regular attendance at a local place of worship or travelling, often internationally, on a pilgrimage. Such meetings are often intergenerational and place people in close proximity, making them a particular focus of spread (12). Other religious assemblies may centre around pivotal life events or rites of passage including births, marriages and deaths, and distinctive religious celebrations such as baptism, confirmation and first communion in Christian churches.

Religious assemblies such as funerals were temporarily banned for COVID-19 reasons, yet large numbers of individuals were reported to have gathered for mourning at Hasidic Jewish funerals in New York and London in the spring of 2020 (13). Given the importance of gatherings, including for the purposes of worship, to religious believers and the fact that in the UK, Muslims and those of some other faiths are over-represented in areas of material deprivation, it is not surprising that in the UK a number of religious communities have been disproportionately affected by COVID-19. Data taken from the 2011 UK Census (the most recent available), alongside those of registered deaths due to COVID-19, show that the ‘highest age-standardised mortality rates (ASMRs) of deaths involving COVID-19 were in the Muslim religious group with 198.9 deaths per 100,000 males and 98.2 deaths per 100,000 females (p.3); people who identified as Jewish, Hindu or Sikh also showed higher mortality rates than other groups’ (14).

In the same report, those who reported ‘no religion’ had the lowest rate of death involving COVID-19, with 80.7 deaths per 100,000 males and 47.9 deaths per 100,000 females. The report explained that, for the most part, these elevated risks for certain religious groups can be explained by ‘geographical, socio-economic and demographic factors and increased risks associated with ethnicity’ (14, p. 9). For example, in the UK, Southeast Asians are more prone to having type 2 diabetes, a risk factor for mortality from COVID-19, and are more likely to live in large, intergenerational families, which increases the likelihood of COVID-19 transmission (14). It is important to emphasise that in general, religious faith is associated with enhanced health, principally as a result of the social support mechanisms provided by religious groupings (15).

## Religion should not necessarily be seen as a problem

During the COVID-19 pandemic, religion has frequently been depicted as a problem. There is a narrative that sometimes implies (or even states) that religion is either directly harming public health or indirectly undermining the public health response to COVID-19. Noteworthy media headlines have included: ‘The road to coronavirus hell was paved by evangelicals’ from *The New York Times* (16); ‘US churches and pastors ignoring “stay-at-home” orders’ from *The Guardian* (17) and ‘Fears of spike in coronavirus during Ramadan’ from *The Metro* (18). In the case of the Hasidic Jewish funeral in New York mentioned above, the large group of mourners gathered for the funeral at a time when the city was under lockdown and any gatherings were prohibited due to COVID-19 prevention efforts. The New York City mayor Bill de Blasio publicly complained about the congregation (13) and the crowd was dispersed by the New York Police Department, using water cannons.

Some faith communities have experienced immense discrimination. Anti-Semitism, Islamophobia and structural racism can lead communities to feel alienated, even persecuted. Such feelings do not bode well for state–community engagement in public health, nor for trust in local health officials. Crucially at this watershed moment, following the murder of George Floyd in the United States of America (19) and the growth of the Black Lives Matter movement (20), the impact of persistent, long-term structural and institutional racism as a determinant of health, including the health of BAME (Black, Asian and Minority Ethnic) individuals and communities, should be recognised. Such inequalities affect the communities that have fought long and hard in the battle for social justice. This historical context therefore provides a justification as to why representatives of such groups should be involved in community engagement and health policy decisions.

In the Western secular world, religion in its broader sense is often seen as hypocritical, corrupt and the underlying cause of many of today’s conflicts (21). The role of religion in health and development is often seen as fraught with tensions and problematic, in part due to the narratives of missionaries who came to serve but also to proselytise, often with a background of colonialism and racism. There is also a more recent history of scepticism about religious involvement in healthcare and public health due to the overt role religion has played in influencing certain health policies, such as in the case of PEPFAR (The President’s Emergency Plan For AIDS Relief). Here, conservative Christians in the US ensured that funds from the programme were tied to initiatives such as abstinence-only sex education and could not be used for abortion counselling (22). In addition, some scholars have suggested that religion is seen as an ‘anti-development force’ (23) and has had an overall negative contribution to health and development (24). Nevertheless, while religion can often be seen as a problem, both in general and with regards to COVID-19, there are examples of the positive contribution that religion has made in health in the recent past.

## Valuable health promotion lessons from religion

Religion has been used as an important tool to tackle and help reduce the spread of infectious diseases including polio, HIV and Ebola. Obregón and colleagues analysed attempts at eradicating polio in India and Pakistan. Here, most polio cases were amongst young children in predominantly poor Muslim communities and vaccine hesitancy was an issue (25). Some misconceptions about the vaccine were that it was ineffective, harmful to children, caused infertility and represented a deliberate plan to reduce the Muslim population. Muslim religious leaders were asked to participate in the campaign to eradicate polio and, as a consequence, vaccine coverage increased.

In the case of HIV, there have also been examples of working with religious leaders to reduce stigmatisation of those with HIV and utilising religious doctrines to justify a health policy and engage with faith-based organisations. For example, in Malaysia, religious leaders were trained to educate other religious leaders and congregants to increase compassion for those living with HIV and given practical guidance on how to perform Islamic burials for those who had died from AIDS, at a time when some mosques had refused to bury the deceased due to mistaken fears of contracting the virus (26). In South Africa, Ethiopia and Uganda, religious leaders who were themselves living with HIV set up faith-based organisations aimed at reducing stigmatisation, increasing awareness and promoting safer behaviour practices (27).

A recent example of health promotion benefiting from religion was in stopping the Ebola epidemic in West Africa. It became apparent that new Ebola infections were largely due to exposure during funerals and burials. A concerted effort was therefore made to engage with community-based organisations to understand the local context, including religious and cultural aspects that had previously been overlooked (28). Involving Muslim and Christian religious leaders and thereby changing religiously inspired funeral and burial practices proved critical. This example may be particularly relevant to COVID-19, given reports of its spread in some parts of South Africa due to funerals (29) and of cadavers being stolen in hospitals in Indonesia (30) due to fears that the deceased will not be buried in accordance to Islamic protocol.

In general, and particularly in health emergencies, working with religious communities is often undertaken by working through religious leaders. Nevertheless, there is great value in working directly with individuals at all levels of communities (23). In addition to empowering individuals, this can help embed change.

## Incorporating religion within public health

If religion is to be taken seriously within public health, both in relation to COVID-19 and more generally, there are two frameworks which may be useful. The first is complexity theory, which is already being increasingly used in health promotion and the sciences more broadly. The second consists of educational approaches that advocate sensitivity as a means of reducing the chances of conflict when seeking to work with people who hold very different views.

Complexity theory can be understood in a number of ways (31). Its utility for the medical sciences is that it sees systems as open with ‘fuzzy’ boundaries. It takes reflexivity seriously in those objects and processes are seen as being defined through their interactions, and in appreciating both turbulence and unpredictability it is more likely to draw on lessons from mixed-methods case studies than rely on randomised controlled trials alone. These features make it easier to incorporate religion and help health promotion examine the multifaceted ways in which religion may impact health.

In science education, it has been argued that contentious topics such as evolution and vaccine hesitancy can be more effectively communicated to individuals who are disposed to reject them by reframing and considering such positions as worldviews, rather than as manifesting misconceptions, and by treating such individuals sensitively (32). The attempt is not to ‘convert’ a student who comes from a family and/or community background that rejects evolution or the use of vaccines into a student who now accepts them. Rather, it is for a science teacher to realise more sensitively the ecosystem within which the learner sits (not just the science classroom) and for the learner to come to an understanding of currently accepted scientific theories.

What we therefore advocate, by way of operationalisation, is that health promotion incorporates religion within existing (albeit as yet rarely used) frameworks that draw on complexity theory (31), and does so in a way that treats religious considerations sensitively. One way forward is to acknowledge that religions may be understood as worldviews (33), where one person’s worldview may be different from another’s and yet we all want and need to live together in a single world, whether that world is affected by COVID-19 or not.

## Religion for public health

Religion is therefore an important factor to take into account in health. Religion is intertwined in complex ways with individual health behaviour, service delivery, health policies, process and power, and also social norms, beliefs and worldviews, all of which affect health outcomes, either directly or indirectly (6). In a number of countries, especially in the West, religiosity, as typically measured by attendance at worship, is in decline (34). At the same time, the countries in the world where religiosity is increasing tend to be the ones with the highest rates of population growth rate. The projection of the Pew Research Center is that the percentage of the world’s population who are religious will increase slightly from 84% in 2015 to 87.5% in 2060 (35). Furthermore, there is some evidence that in countries where religion is in decline, more people report that they are ‘spiritual’ (34).

Religious beliefs often manifest as powerful and coherent forces that are embedded within every aspect of believers’ lives. Religious beliefs influence what is believed to be of ultimate value, what is eaten and drunk, what clothes are worn, how days are structured, what work is undertaken, who is married and how children are raised (26). Those who are vulnerable are often more likely to manifest religious beliefs or practices. For example, the onset of the COVID-19 pandemic was associated, in the UK, with 5% of adults saying they had started to pray during the lockdown but didn’t pray before, increasing the figure of adults who said that they prayed regularly (at least once a month) from 21% to 26% (36).

Faith-based organisations make a substantial contribution to health services worldwide, especially in low-income countries, with some estimates of their input reaching 50% (37). Churches have a long history of involvement in building hospitals and schools and providing health services. However, there is also a panoply of lesser-known organisations that are religiously motivated, such as the Tzu Chi Buddhist organisation that operates internationally (38). Religion can often influence health policy in countries where there are strong religious affiliations (37). Religious conceptions of health – for example, seeing good health as a gift from God – frequently influence health practices and behaviours (6). Religious organisations and religious leaders often influence health policy, and religious groups can have significant lobbying power on governments. At a more grass-roots level, religious leaders individually often play vital roles and exercise moral authority within communities. They can hold a great wealth of local knowledge, have extensive networks and be trusted (something that is hard to quantify), and are important sources of pastoral support and guidance (26).

The African Religious Health Assets Programme (39) attempted systematically to map out the many valuable assets that religions may be able to contribute in the religion–health field. It concluded that many of these could be leveraged for better health and development. Tangible assets include places of worship, other facilities and general infrastructure. Other assets include a religion’s ability to motivate and mobilise communities in shared causes and to engender compassion, purpose and hope.

Most religions promote well-being and health in their scriptures and other teaching, and place a high priority on the sanctity of life. In fact, many religious prohibitions, for example, on drinking alcohol, gambling, drug taking, gluttony and suicide, are influenced by beliefs about the importance of the human body (seen as a temple of the Holy Spirit in Christianity) and life in general (11). While a more pathogenetic focus on disease is predominant in medicine and even public health, religion could be used to advocate for a shift in focus to a more ‘salutogenic’ (40), health-promoting approach. For example, ideas of physical purity and cleanliness, with ‘Cleanliness is next to Godliness’ (a phrase popularised by the Methodist leader John Wesley in the late eighteenth century (41)), could promote handwashing, a key strategy in the defence against COVID-19.

In many places, individual religious leaders have a pivotal role acting as ‘gatekeepers’. They may disseminate health information, or allow health professionals to come into places of worship and deliver such information in a sermon or address. These religious leaders can often reach those who are marginalised, such as migrants, those who cannot readily access health information though conventional routes, because of language barriers, and the elderly. Furthermore, they can directly disseminate information from the ‘pulpit’ to their congregants and can promptly address misinformation regarding, for example, erroneous conspiracy theories that COVID-19 is a hoax, or that vaccines are a weapon of colonialism. They are able to communicate messages in a way that does not cause harm, always important when trust in science is weak, but imperative during a pandemic (42).

Religion is often seen as an important coping mechanism, especially during times of stress and anxiety. It can offer perspective, hope and meaning in uncertain times, which can act as an anchor for those predisposed to mental health issues. During the COVID-19 pandemic, because many places of worship were required to close, many religious bodies adapted their patterns of worship and moved from face-to-face to online services (43). Religious groups can make decisions regarding whether pilgrimages or other events should take place. For example, in Saudi Arabia, the 2020 Hajj has, to all intents and purposes, been cancelled, except to a small number of Saudi residents (44). Religious leaders can sometimes make decisions more expeditiously than governments. Religious leaders can also facilitate the implementation of health policy. For example, after attendants at a religious gathering in Malaysia failed to come forward to be tested, the Minister for Religious Affairs issued a statement saying that it was a religious obligation to be tested (45).

Van den Broucke (46) succinctly articulated how ‘Enabling people to increase control over their health and its determinants is at the core of health promotion’ (p.181). Drawing on Brownson et al. (47), Van den Broucke goes on to argue that health promotion can address COVID-19 at three levels: the downstream level, focussing on individual behaviour change and disease management; the midstream level, through interventions that operate on organisations and communities; and the upstream level, through changing policies that affect whole populations. As the examples in this section show, religion can influence all three levels and so contribute to the management of COVID-19. Marston et al. (48) demonstrated that pandemic responses have traditionally been top-down, with governments imposing policies on communities, and therefore allowing little or no input from these communities. However, such top-down approaches represent a wasted resource. Communities are not only knowledgeable about on-the-ground realities, they may be able to identify solutions, insights and potential barriers and co-design effective responses. This is particularly important for unpopular measures that risk low compliance. Religious organisations are well placed to offer services which address the indirect effects of COVID-19, such as deteriorating mental health, isolation and a rise in the frequency of domestic violence, given the pastoral nature of religious duties.

## Discussion

There are a number of overarching issues that follow from our analysis which are important for moving forward with the COVID-19 response internationally. There should be a greater appreciation of the role of religion and belief in public health, particularly when attempting to understand social determinants of health in their contexts. Health professionals are encouraged to ensure that communication with the public is culturally and religiously sensitive, is appropriately targeted at specific communities, and does not apportion blame. While health systems are complex, particularly when trying to take account of religious sensitivities in a pluralist world, health promotion is possible at a range of levels. This may appear a rather modest recommendation, yet treating people sensitively is a key part of listening to them and appreciating that the way they see the world (their worldviews) may be different from one’s own. One’s own viewpoint is not necessarily superior and, anyway, one’s own way of understanding reality may not translate to the contexts in which others find themselves.

Complexity theory takes reflexivity seriously and objects and processes are seen as being defined through their interactions. Religion and health can be understood in this way – not only do they interact but, to a believer, conventional measures of good health may not always be what is most important. For instance, those with a religious faith may understand the physical health risks of meeting with others but still wish to attend communal worship on a regular basis. We can learn from past successes and failures in public health and understand how attempts were made to deal with health issues and what mechanisms were employed. It is also possible to analyse how possible conflicts were anticipated and sometimes managed successfully. For example, in some countries in the East, the HPV vaccine has avoided the parental hesitancy and reluctance which have been apparent in some countries in the West by reframing the inoculation as a ‘cancer-preventing vaccine’. Such reframing has increased buy-in and engagement with communities, within cultural and religious beliefs, and led to greater vaccination uptake in schools (49).

Understanding religion within the context of the societal norms that operate within communities can help us to be mindful of the power dynamics and better understand health behaviours. Michie and colleagues explain the importance of understanding health behaviour in order to slow down COVID-19 and offer a five-point plan: creating a mental model; creating social norms; creating the right level and type of emotion; replacing one behaviour with another; and making the behaviour easy (50). Mitchie et al. pointed out that replacing a behaviour with another behaviour is often more effective than simply telling people to stop the offending behaviour. With regards to COVID-19, this means, for instance, that it may be helpful to advise people to keep their hands beneath shoulder level, rather than simply telling them not to touch their face.

Mitchie et al.’s approach to COVID-19 prevention could be enhanced with religious-informed additions. For example, in the case of mask-wearing, the wearing of masks can be made easier by supplying them at places of worship if people forget to bring their own. Creating the right level and type of emotion can be facilitated by explicitly addressing religious beliefs about fatalism or protection. New behaviours can be embedded within existing routines that are rooted in religious practice, such as the washing of hands. Prominent influencers in the community, such as religious leaders, can wear masks themselves and urge congregants to wear them, so that this becomes the new social norm. Hong and Handal (51) argue that given the current crisis there is a need to engage different institutions, religious groups, civil society and the government. Furthermore, measures should not simply be imposed on faith groups; rather, faith groups should be involved in decision making from the start as *bone fide* stakeholders, as recommended in the Public Health England report on the disparities in the risk and outcomes of COVID-19 (52).

Overall, attempts at controlling infectious diseases have not always made best use of lessons from health promotion, with its rich theoretical underpinnings in both medicine and the social sciences (53). In so far as COVID-19 goes, we simply do not have the luxury to not take advantage of all that we know about human behaviour in tackling disease. If religions are treated sensitively, with an acknowledgement of the complexities of the issues and the need for solutions at a number of levels, there is a good chance that some of the worst predictions of the consequences of COVID-19 may not come to pass. But if religions are ignored or belittled (54), COVID-19 could continue unabated in some religious communities, thereby broadening the impact of this global pandemic.
